# Network Pharmacology in Traditional Chinese Medicine

**DOI:** 10.1155/2014/138460

**Published:** 2014-02-24

**Authors:** Shao Li, Tai-Ping Fan, Wei Jia, Aiping Lu, Weidong Zhang

**Affiliations:** ^1^Bioinformatics Division, TNLIST/Department of Automation, Tsinghua University, Beijing, China; ^2^Department of Pharmacology, University of Cambridge, Cambridge, UK; ^3^University of Hawaii Cancer Center, Honolulu, USA; ^4^School of Chinese Medicine, Hong Kong Baptist University, Hong Kong; ^5^Department of Pharmacy, Second Military Medical University, Shanghai, China

Network pharmacology is becoming a cutting-edge research field in current drug discovery and drug development thanks to rapid progress in systems biology, network biology, and chemical biology. By integrating reductionist and systems approaches as well as computational and experimental methods, network pharmacology has great potential to act as the next generation mode of drug research. Network pharmacology studies emphasize the paradigm shift from “one target, one drug” to “network target, multicomponent therapeutics,” highlighting a holistic thinking also shared by traditional Chinese medicine (TCM). In TCM, the perspective of holism has long been central to herbal treatments of various diseases. Characterized by holistic theory and rich experience in multicomponent therapeutics, TCM herbal formulae offer bright prospects for the control of complex diseases in a systematic manner. Thus, introducing network pharmacology in TCM will provide novel methodologies and new opportunities for discovering bioactive ingredients and endogenous/exogenous biomarkers, revealing mechanisms of action and exploring scientific evidence of numerous herbs and herbal formulae in TCM on the basis of complex biological systems of human body. Moreover, the integration of TCM and network pharmacology can greatly promote the progress of network pharmacology as well.

Here, we have grouped together 27 papers in this burgeoning field, put forward for publication in this special issue on TCM network pharmacology.

In the special issue, we have firstly published four concise reviews or perspectives across the two fields between TCM and network pharmacology. The topics range from the research paradigm of network pharmacology based on TCM theory and practice, the available databases and computational tools in TCM network pharmacology, to the applications of network pharmacology in TCM. These papers highlighted some specific themes, such as the concept of network target, mechanisms of TCM herbal formulae, and target identification of herbal active ingredients. For example, a review article provided a perspective regarding TCM-based network pharmacology and its use in multiple compound drug discovery, by following an analysis of the merged networks of differentially expressed genes in rheumatoid arthritis-cold pattern and protein targets related to Fu Zi, Xi Xin and Gui Zhi. Three other review articles comprehensively addressed the origin and development of TCM network pharmacology, the definitions of basic network concepts, the computational tools and data sources regarding TCM network pharmacology, and the significance and approaches of network pharmacology in the TCM field, as well as the target identification methods of herbal active ingredients and the use of ligand-protein networks.

A remarkable feature of TCM is the use of herbal formulae. Understanding the mechanisms of action and combinatorial rules of herbal formulae is of great significance in TCM modernization and is also one of the important steps in modern drug discovery. The emerging TCM network pharmacology offers a unique opportunity to explore systematically not only the molecular complexity of an herbal formula, but also the molecular relationships between an herbal formula and complex diseases. A practical strategy of TCM network pharmacology is the combined use of network-based computational predictions and experimental validations. In this special issue, we have published 11 interesting research papers covering 10 classic herbal formulae by employing network-based approaches and omics-based experimental studies. For example, two research papers established an integrative platform of TCM network pharmacology on the basis of the concept of “network target, multicomponent therapeutics,” and then applied this platform in the identification of active compounds and mechanisms of action of an herbal formula *Qing-Luo-Yin* for the treatment of rheumatoid arthritis, as well as *Ge-Gen-Qin-Lian* decoction recorded in “*Shang-Han-Lun*” for the treatment of Type 2 diabetes. Moreover, network analysis and omics technologies including genomic, metabolomic, and proteomic studies are always integrated together to understand the molecular actions exerted by herbal formulae at a systematic level. In the special issue, four papers addressed the metabolomic analysis for determining the effects of *Huang-Lian-Jie-Du-Tang* in a rat model of collagen-induced arthritis, the metabolomic analysis for *She-Xiang-Bao-Xin* pill in treating myocardial infarction in rats, the proteomic analysis for determining the possible proteomic network associated with the antiarthritic effects of *Qing-Fu-Guan-Jie-Shu* in collagen-induced arthritis rats, and the experimental study of the protective effect of *Xiao-Xu-Ming* decoction in a rat model of cerebral ischemia and reperfusion. It is known that identifying the target proteins and combinatorial rules of active ingredients in herbal formulae remains to be a difficult issue. There are six papers to address such an issue from the network point of view by using bioinformatics analysis and experiments, for example, the mechanism of antirheumatic actions of *Huang-Lian-Jie-Du-Tang *by network analysis, the molecular mechanism of *Shu-Feng-Jie-Du* formula by a module analysis, drug-target network of *Yuan-Hu-Zhi-Tong *prescription, the pharmacological mechanisms of *Wu-Tou-Tang*, and the mechanism of cell apoptosis induced by *Fu-Zheng-Hua-Yu* tablet.

Herbal active ingredients have long been viewed as a rich source of therapeutic leads in drug discovery. Network pharmacology is expected to be a new strategy and powerful tool to find the bioactive compounds as well as their potential molecular targets from numerous herbs or herbal formulae. For herbal active ingredients, there are eight papers published in this special issue with the efforts to study herbal active ingredients by network pharmacology approaches. For example, a paper predicted the target network of vitexicarpin and experimentally validated the molecular network account for its antiangiogenic activities. A paper proposed a computational method to identify the effective herbal components and mechanisms of action. A paper revealed a pharmacological signaling pathway network of baicalin in the treatment of ischaemia-reperfusion in mice. Three other papers addressed the molecular mechanisms of herbal active ingredients with the help of omics technologies, including the metabolomic analysis of genipin in rats and identification of metabolites by LC/MS, the neuroprotective effects of hydroxysafflor yellow A via the NF-kappaB pathway by NMR-based metabonomics, and expression profiling and proteomic analysis of JIN Chinese herbal formula in lung carcinoma H460 xenografts. Lastly, two experimental studies are published on the epithelial-mesenchymal transition induction of shikonin in skin wound healing and capsaicin-induced cancer cell apoptosis through ER stress.

Uncovering the scientific basis of herbal traditional properties especially the molecular association between herbal formulae and TCM syndrome (ZHENG) is absolutely critical to preserve and develop TCM. Four papers in this special issue focus on the traditional properties of TCM. A paper identified the network-based biomarkers for the Cold Coagulation Blood Stasis Syndrome and showed the therapeutic effects of *Shao-Fu-Zhu-Yu* decoction in rats. To identify potent synergistic combinations in herbal formulae, a paper developed an interesting Feedback System Control Scheme to optimize combinations of flavonoids derived from Astragali Radix. As the potential adverse effects of herbs have limited their clinical applications, a paper proposed a network-based computational model to predict the safety of herbs through a comparative analysis of withdrawn drugs. Based on combining formula network, chemical space, and metabolic space, a paper analyzed the properties of the incompatible herbal pairs associated with the traditional rule termed “eighteen antagonisms and nineteen mutual inhibitors” in herbal formulae.

In summary, TCM network pharmacology is a new interdisciplinary frontier in both ancient TCM and modern drug discovery and development fields, which represents valuable interactions and exchanges between traditional Chinese medicine and those of network, pharmacological, biomedical and computational sciences. This special issue provides a high-profile venue for dissemination of significant scientific findings in TCM network pharmacology. It is just the beginning. We encourage researchers in TCM and related fields to support the development of this novel and promising direction.

*Shao Li* *Shao Li* *Tai-Ping Fan* *Tai-Ping Fan* *Wei Jia* *Wei Jia* *Aiping Lu* *Aiping Lu* *Weidong Zhang* *Weidong Zhang*

